# Transcutaneous electrical stimulation of the stellate ganglion: A case report on its application in treating carotid sinus syndrome

**DOI:** 10.1097/MD.0000000000039388

**Published:** 2024-09-06

**Authors:** Taifu Hou, Mengya Xu, Zhiguo Zhang

**Affiliations:** a Wushu College of Henan University, Kaifeng, China; b Department of Neurological Rehabilitation, The Second Affiliated Hospital of Zhengzhou University, Zhengzhou, China.

**Keywords:** carotid sinus hypersensitivity, carotid sinus syndrome, stellate ganglion, syncope, transcutaneous electrical nerve stimulation

## Abstract

**Rationale:**

Syncope is a common condition in emergency departments, posing a diagnostic challenge due to its multifactorial nature. Among the potential causes, carotid sinus hypersensitivity leading to carotid sinus syndrome (CSS) is a significant consideration that can severely impact patient quality of life. Despite its importance, establishing effective treatment methods for CSS has been difficult.

**Patient concerns:**

A 43-year-old male presented with recurrent episodes of syncope, which significantly affected his daily life and well-being.

**Diagnoses:**

After a thorough evaluation, the patient was diagnosed with CSS, a condition that can be difficult to pinpoint and requires specialized diagnostic procedures to confirm.

**Interventions:**

The patient was treated with stellate ganglion block therapy, a targeted intervention aimed at addressing the underlying cause of CSS. This treatment was administered over a 12-day period.

**Outcomes:**

Following the treatment, the patient’s symptoms showed gradual improvement, and he was discharged after meeting the clinical cure criteria. During a 7-month follow-up, he remained symptom-free.

**Lessons:**

The case highlights the effectiveness of transcutaneous stellate ganglion block therapy in treating CSS. It suggests that further research and clinical trials are needed to validate this treatment’s efficacy, potentially offering a new therapeutic option for patients suffering from CSS.

## 1. Introduction

Syncope, marked by sudden loss of consciousness and global cerebral hypoperfusion that results in an inability to maintain posture and muscle tone, frequently prompts emergency department visits.^[1]^ It presents a complex and pervasive challenge in healthcare, with symptoms that vary widely. Despite thorough and costly investigations, the causes and underlying mechanisms often remain elusive.^[2]^ The carotid sinus, a neurovascular structure containing baroreceptors, is situated at the point where the internal and external carotid arteries merge into the common carotid artery. This structure, which experiences a dilation at the bifurcation of the carotid arteries, can exhibit carotid sinus hypersensitivity (CSH) when its receptors become overly reactive to stimulation.^[3]^ This hypersensitivity often leads to syncope and primarily affects older adults and those with cardiovascular, cerebrovascular, and neurodegenerative conditions.^[3]^ Although CSH is not a standalone clinical diagnosis, it can manifest as carotid sinus syndrome (CSS) in some individuals. CSS, a poorly understood autonomic nervous system disorder, causes recurrent unexplained syncope and frequent falls.^[4]^ The origins of CSS are mostly unknown, and epidemiological data remain scarce,^[5]^ significantly impairing patients’ quality of life and causing substantial physical and psychological distress. In this particular case, the patient with CSS received transcutaneous electrical nerve stimulation (TENS) at the stellate ganglion, which will be detailed in subsequent reports.

## 2. Clinical data

The patient, a 43-year-old male, presented at the rehabilitation clinic on July 10, 2022, reporting “recurrent unexplained dizziness and even syncope over the past 2 years, worsening in the last 6 months.” Fifteen years earlier, he had undergone microsurgical replantation of the distal joint of his right index finger following trauma in Shenzhen, though the specific hospital and details of the surgery are not known. The surgery successfully restored full functionality to his hand. He reported no history of blood transfusions or donations and confirmed adherence to local vaccination protocols. He also denied any history of allergies.

Blood pressure was measured at 125/82 mm Hg and heart rate at 85 beats/min. During a specialized examination, the patient’s cervical curvature was normal; there were no significant changes in local skin color or temperature, and no palpable nodules, rashes, or indurations were present. There was no tenderness when palpating over the C4/C5/C6/C7 spinous processes or adjacent paravertebral muscles. Pain on forward neck flexion and tenderness at the exits of the greater occipital nerves bilaterally were noted. Nevertheless, neck mobility was functional, exhibiting normal flexion, extension, lateral flexion, and rotation. Sensory tests confirmed intact skin perception, and muscle strength was symmetrical with no signs of atrophy in all limbs; tendon reflexes were normal, with negative results for Hoffman sign, positional vertigo, head compression test, Spurling test, cervical torsion test, and neck extension test.

Imaging examination: a chest X-ray and electrocardiogram (dated June 29th) showed no abnormalities. A computed tomography scan of the cervical spine, also conducted on June 29th, displayed normal cervical lordosis without any other significant findings. A color Doppler ultrasound of the bilateral neck vessels revealed no noticeable abnormalities in the carotid arteries (venous) on either side. Routine laboratory biochemical tests were within normal limits.

The patient shared additional historical details, noting episodes of dizziness, chest tightness, and a feeling of suffocation when a barber bib was placed around his neck. These symptoms would subside upon removal of the bib. Additionally, the patient reported a 2-year history of avoiding high-collared clothing due to the onset of similar symptoms, including dizziness, tinnitus, chest tightness, and a sensation of suffocation. Similar symptoms occurred when a quilt was wrapped around his neck at night, alleviating upon loosening his collar or pulling down the covers.

**Figure 1.**
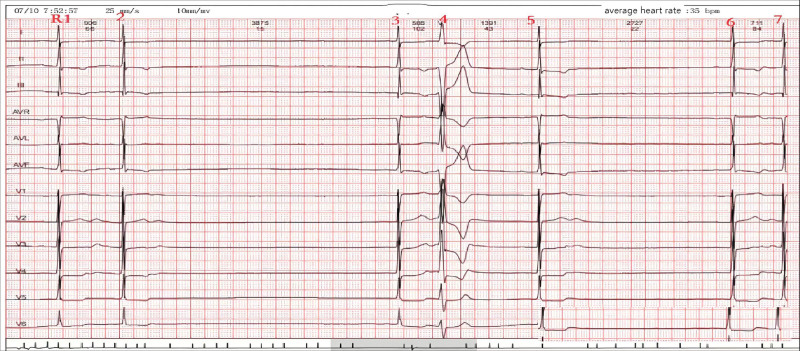
ECG recorded during the initial carotid sinus stimulation test.

Differential diagnosis included assessing psychogenic factors, where the Somatic Symptom Scale yielded a score of 16, the Generalized Anxiety Disorder 7-item scale registered a score of 3, and the 9-item Patient Health Questionnaire also scored 3, effectively ruling out psychological causes. A Head-Up Tilt Test was administered with initial blood pressure readings at 125/82 mm Hg. During ECG-monitored carotid sinus massage, the patient experienced symptoms of dizziness, chest tightness, and syncope, with blood pressure dropping to 70/42 mm Hg and ECG showing a ventricular arrest lasting 3.9 s (≥3 s), as depicted in Figure 1. The systolic blood pressure decreased by 55 mm Hg (>50 mm Hg), leading to syncope and generalized weakness. These symptoms aligned with a diagnosis of CSS.^[2]^

**Figure 2.**
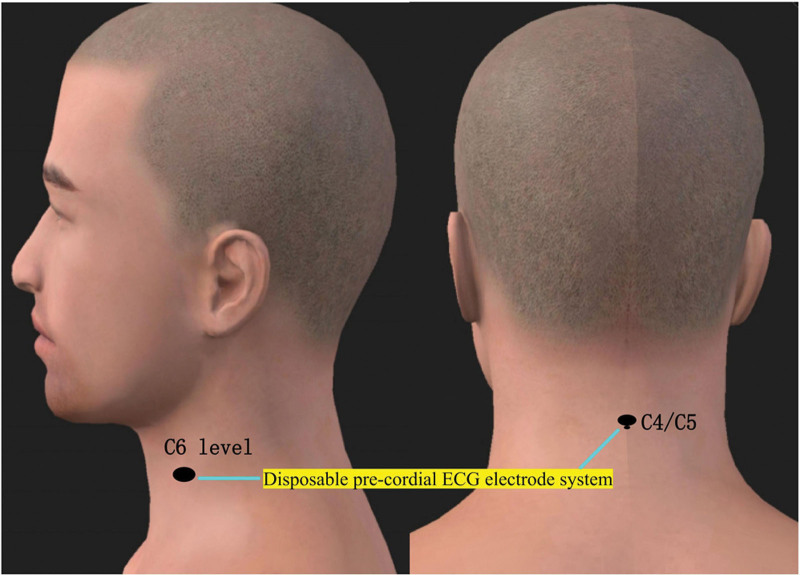
Diagram illustrating the application site of transcutaneous electrical nerve stimulation (TENS).

To treat the patient’s condition, we employed physical factor therapy using TENS, specifically targeting the stellate ganglion with a WOND2000F2 Multifunctional Neurological Rehabilitation Diagnosis and Treatment System (Guangzhou Sanjia Medical Equipment Co., LTD). We set the stimulation parameters to a frequency of 100 Hz and a pulse width of 100 μs, adjusting the current intensity to the patient’s comfort level, measured in milliamps (mA). We followed standard disinfection procedures using 75% alcohol to ensure cleanliness and reduce biological impedance. Two disposable ECG electrodes were placed on the patient’s body: one on the body surface projection at the midline of the C4/C5 vertebrae on the patient’s back, and the other on the left anterolateral surface projection of the stellate ganglion, which previous studies suggested is more effective than the right side.^[2]^ The electrodes were aligned parallel to the C7 vertebra throughout the treatment, as shown in Figure 2. The treatment sessions were conducted twice daily, in the morning and afternoon with a 6-hour interval, each lasting 20 minutes, over a 12-day period.

### 2.1. Treatment results

**Figure 3.**
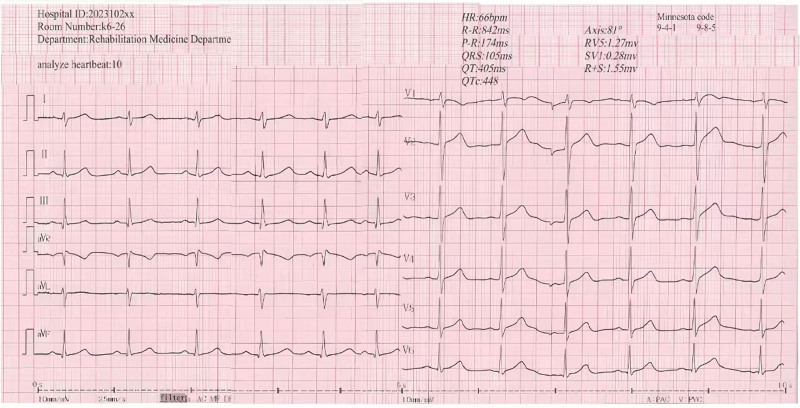
ECG recording during the second carotid sinus stimulation test.

Three days into the treatment, the patient experienced only mild dizziness when touching his neck. By the 6th day, he could wear high-necked clothing without suffering from dizziness, tinnitus, chest tightness, a sense of asphyxia, or fatigue. On the 7th day, after resting, he was able to slightly tighten his high-necked clothing and pull the covers closer to his neck during sleep without any discomfort. By the 10th day, he could gently grasp his neck with his hand without any discomfort. On the 12th day of treatment, his blood pressure was recorded at 127/83 mm Hg, remaining stable at 122/81 mm Hg after a carotid sinus stimulation test, with the ECG indicating a ventricular arrest of approximately 0.78 seconds (see Fig. 3). Following 2 additional days of consolidation treatment, the patient fulfilled the clinical cure criteria and was discharged. During monthly follow-ups over the next 7 months, the patient did not experience any recurrence of syncope in his daily activities.

## 3. Discussion

After more than 50 years of studying the physiology and pathology of the carotid sinuses, researchers have established that: (1) the carotid sinus functions as a baroreceptor sensitive to the stretching of the arterial wall. When external stretching stimuli exceed a certain threshold, it triggers increased activity in the vagus nerve and decreased activity in the sympathetic nerve. In contrast, a drop in arterial blood pressure leads to decreased blood flow and vessel wall stretching, which in turn reduces the baroreceptors’ firing rate and lowers vagus nerve excitation. The afferent pathway of this baroreceptor-mediated reflex involves the glossopharyngeal and vagus nerves, which transmit impulses from the carotid sinus to the brainstem, ending in the solitary tract nucleus in the medulla oblongata. The reflex’s efferent signals are then relayed to the heart and blood vessels through the sympathetic and parasympathetic nerves (vagus nerves), regulating heart rate and vasomotor tone. This impulse transmission via the vagus nerve is crucial in the development of CSS.^[6]^

Stellate ganglion block involves administering a local anesthetic near the stellate ganglion to disrupt the conduction of sympathetic nerve impulses. This therapeutic approach aims to correct dysfunction in the autonomic nervous system and promote stability in the body’s internal environment.^[7]^ Research indicates that noninvasive stellate ganglion block can be achieved through physical therapy techniques,^[8]^ which have been shown to effectively reduce cardiovascular sympathetic activity, stress responses, and vascular tone, ultimately improving vascular function in the limbs.^[9]^

TENS is a therapeutic approach that delivers pulsed alternating current to a specific treatment area via electrodes placed on the skin.^[10]^ Studies have indicated that positive membrane potentials occur in fibers N1 and N3 at depths ranging from approximately 21 mm to 23 mm, which coincides with the typical needle depth for stellate ganglion nerve block (2.0–2.5 cm).^[11]^ The mechanism of action is rooted in the gate control theory, which suggests that activating large nerve fibers (A-β fibers) and exciting glial cells (Stellate Ganglion Cells) in the spinal dorsal horn can inhibit the transmission of nociceptive information through extraganglionic analgesic mechanisms.^[12]^

In this case, the chosen stimulation parameters were a high frequency of 100 Hz and a pulse width of 100 μs. Peripheral blockade induced by TENS within thick fibers can create a “busy line effect,” causing collisions between nerve impulses in A-δ fibers and nociceptive impulses from injured tissues. This phenomenon primarily involves segmental mechanisms, potentially leading to segmental inhibition of nociceptive information transmission in the spinal dorsal horn, thereby reducing afferent impulses from the periphery and lowering nerve sensitivity to achieve therapeutic effects for CSH.^[13]^ Pfyffer et al found that high-frequency TENS could stimulate A-β fibers and activate Stellate Ganglion cells to release inhibitory neurotransmitters, thereby suppressing damaging sensory signals generated by the excitatory action of projective neurons on the same segmental sensory fine fibers in the spinal cord dorsal horn.^[10]^ Additionally, TENS can produce thermal effects that decrease the excitability of sensory nerves, further contributing to treatment efficacy.^[14]^ Recent studies have suggested that TENS can influence sensory nerve thresholds, as evidenced by current perception threshold measurements conducted below the threshold level. It was observed that A-β fibers may exhibit lower current perception threshold values with subthreshold TENS, indicating that this type of stimulation may selectively enhance A-β fiber function.^[15]^ By reducing neural sensitization and decreasing sensory fiber excitability, TENS can effectively address CSS. Moreover, TENS increases Golgi organ tension and reduces resistance caused by the viscoelastic properties of nerves and muscles.^[16–18]^ This significantly improves muscle flexibility, autoinhibitory reflexes, strength, balance, and proprioception, while effectively alleviating muscle fatigue, which is considered a crucial factor affecting voluntary muscle control, posture, and balance.^[17]^

In this instance, the pre- and post-treatment data from carotid sinus stimulation illustrated the efficacy of percutaneous stellate ganglion stimulation in managing CSS, as outlined in Table 1.

**Table 1:** Changes in data observed during the carotid sinus stimulation test before and after treatment.

Before treatment				12 days after treatment			
BP before test	Systolic blood pressure (SBP)	Diastolic blood pressure (DBP)	Ventricular arrest	BP before test	Systolic blood pressure (SBP)	Diastolic blood pressure (DBP)	Ventricular arrest
125/82 mm Hg	70 mm Hg	42 mm Hg	3.9 s	127/83 mm Hg	122 mm Hg	81 mm Hg	0.04 s

The benefits of this therapy encompass its affordability, non-invasiveness, safety profile, minimal occurrence of serious adverse events, and reversibility of mild adverse effects. Nevertheless, factors influencing its effectiveness, such as precise electrode placement and optimal stimulation intensity, should be taken into account. Enhanced accuracy in electrode positioning and higher current intensity typically yield superior outcomes.^[10]^ However, despite these potential advantages, there is currently limited high-quality clinical evidence supporting this treatment. Further clinical research is warranted to validate its efficacy.

## 4. Conclusion

TENS stands out as an affordable and noninvasive therapy, showing promising potential in treating CSS, as evidenced by this case study. However, while the results are encouraging, further clinical evidence is necessary to substantiate its effectiveness conclusively. Future research endeavors will likely concentrate on refining and tailoring treatment approaches for CSS and related conditions. With ongoing advancements in medical technology and a deeper understanding of neuromodulation mechanisms, we anticipate witnessing significant breakthroughs in clinical interventions based on neuromodulation. This holds promise for improving the management of syncope and related disorders, offering renewed optimism for patients in need of effective treatments.

## Acknowledgments

We are particularly grateful to all the people who have given us help on our article.

## Author contributions

**Conceptualization:** Taifu Hou, Zhiguo Zhang.

**Data curation:** Taifu Hou, Zhiguo Zhang.

**Formal analysis:** Taifu Hou.

**Investigation:** Mengya Xu.

**Supervision:** Mengya Xu, Zhiguo Zhang.

**Visualization:** Mengya Xu.

**Writing – original draft:** Taifu Hou, Zhiguo Zhang.

**Writing – review & editing:** Taifu Hou, Mengya Xu, Zhiguo Zhang.
